# Deciphering genotype‐by‐environment interaction in new soybean lines based on multiple traits using different adaptability and stability methods

**DOI:** 10.1002/fsn3.3996

**Published:** 2024-02-14

**Authors:** Parastoo Majidian, Bahram Masoudi, Ebrahim Hezarjaribi, Nasrin Razmi, Kamal Peyghamzadeh, Amir Gholizadeh

**Affiliations:** ^1^ Crop and Horticultural Science Research Department, Mazandaran Agricultural and Natural Resources Research and Education Center Agricultural Research Education and Extension Organization (AREEO) Sari Iran; ^2^ Seed and Plant Improvement Research Department, Seed and Plant Improvement Institute Agricultural Research Education and Extension Organization Karaj Iran; ^3^ Crop and Horticultural Science Research Department, Golestan Agricultural and Natural Resources Research and Education Center Agricultural Research Education and Extension Organization (AREEO) Gorgan Iran; ^4^ Crop and Horticultural Science Research Department, Ardebil Agricultural and Natural Resources Research and Education Center Agricultural Research Education and Extension Organization (AREEO) Ardebil Iran

**Keywords:** adaptability, AMMI, GGE biplot, pure line, yield

## Abstract

The multi‐environmental trials aid breeders in selecting the best genotypes for specific or general adaptability to different environments before commercial release. This study aimed to assess the stability of 13 new soybean pure lines, along with two controls, in terms of seed yield and important agronomic traits. The assessment was based on a completely randomized block design with three replications across four areas during 2020–2022. Various adaptability methods, including parametric, AMMI, GGE biplot, PCA, and SIIG were employed. The mixed analysis showed that the effects of environment, genotype, and genotype–environment (GE) interaction were significant for most studied traits. The AMMI showed the highest portion of environment (65.89%) in soybean seed yield. Based on all stability parameters, lines 2 and 5 were selected for their average seed yields of 3349 and 3142 kg ha^−1^, respectively. Additionally, lines 6 and 5 showed the most stability, yielding higher than the average, which were 2992 and 3142 kg ha^−1^, respectively, according to GGE biplot results. Furthermore, lines 2, 5, and 8 were identified as the ideal genotypes concerning seed yield and other agronomic traits, with high SIIG parameters and yields exceeding the average. Finally, the soybean line 5 was deemed the most suitable due to its higher yield, stability, and early maturity (128‐day growth period). Therefore, line 5 is considered appropriate for its high stability and earliness in various regions of Iran.

## Graphical abstract

Our findings indicated the soybean line 5 as the most suitable soybean line due to highest yield, stability, and earliest maturity among other lines and cultivars in different regions of Iran using various stability parameters including univariate and multivariate stability parameters.

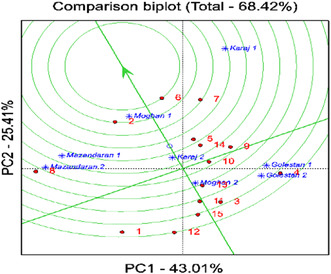

## 1 INTRODUCTION

Cultivated soybean (*Glycine max* L.) is a self‐pollinating crop, known for being a rich source of oil (approximately 20%), protein (around 40%), and carbohydrate (about 35%). Due to its high adaptability, this crop is cultivated across a wide range of latitudes and serves various purposes, including as a food source for humans and animals, and for manufacturing different industrial products (Sinclair et al., 2014). In Iran, leading soybean‐producing areas include Ardebil, Golestan, Mazandaran, Lorestan, and Khuzestan provinces, covering 82,000 hectares and producing approximately 200,000 tons of soybeans, as reported by FAOSTAT statistics (2021). Commercial soybean cultivars in Iran, developed from previous breeding programs, are grown in various regions. Despite the considerable availability of Iranian soybean germplasm, the limited diversity of these varieties does not meet the country's high demand for oil and protein content in human and livestock food rations (Baghbani‐Arani et al., 2021). Consequently, industries related to oil and livestock/poultry production rely heavily on imports of soybean seed and soybean meal. This underscores the urgent need to increase soybean yield and production in Iran.

In recent decades, research has focused on breeding programs to develop new lines/genotypes with higher seed/oil yields, as the right agronomic cultivar is a key factor in successful agriculture. It is vital to produce and use cultivars compatible with changing environmental conditions to achieve optimal yields. The variation of agronomic cultivars in different environmental conditions is, therefore, of great importance. Although researchers have introduced seven new soybean cultivars for different areas in Iran, the existing soybean genotypes lack sufficient genetic variation, limiting farmers' ability to choose the right cultivar for every situation (Nehbandani et al., 2021). Furthermore, the interaction effect of genotype and environment on quantitative traits like seed yield means that genotypes do not yield uniformly across different areas.

In other words, genotype, environment, and genotype–environment interactions all influence each genotype's yield (Siddquie & Hoque, 2023). Understanding genotype–environment interaction effects in breeding programs can assist breeders in identifying yield variations unexplained by individual genotypes and environmental factors (Rani et al., 2021). The presence of stability in a genotype across diverse conditions indicates less genotype–environment interaction. Therefore, modeling genotype–environment interactions in multi‐environmental trials (METs) is crucial for identifying genotypes with general and specific adaptability (Kirankumar et al., 2023).

Statistical analysis methods, such as variance analysis, regression, and instability methods, have been introduced to estimate the main effects of genotype, environment, genotype–environment interactions, and stability determination (Eberhart & Russell, 1966). In most of these methods, however, some basic assumptions of stability analysis are not reliable, including the nonlinear interaction of genotype and environment, and the dependence of the independent variable (environment index) on the function variable (genotype index) (Khan, Kamran Khan, et al., 2021; Khan, Rafii, et al., 2021; Oladosu et al., 2017). In fact, it is feasible to estimate the magnitude and interaction effect of genotype–environment using multivariate methods, which include principal component analysis, additive, and multiplicative effect analysis (Bhartiya et al., 2017; Khan, Kamran Khan, et al., 2021; Khan, Rafii, et al., 2021). Additive main effects and multiplicative interaction (AMMI) analysis is a widely used multivariate stability method for investigating genotype–environment interactions (GEI). This method interprets a large portion of the GEI sum of squares by combining ANOVA for the genotype and environment main effects with principal components analysis (Gauch & Zobel, 1996; Yan et al., 2002).

Among various stability methods, the GGE biplot (genotype main effect plus genotype–environment interaction) serves as a practical graphical tool for modeling genotype–environment interaction in multi‐environment trials (METs) across different crops. The GGE biplot method is capable of identifying the best‐performing genotype in a given environment and the most suitable environment for a specific genotype. Furthermore, this tool assists breeders in determining the relationships between environments and in comparing genotypes based on average yield and stability (Ansarifard et al., 2020; Olanrewaju et al., 2021; Scavo et al., 2023). The use of this method has been widespread in various soybean regions, such as southern Ontario (Yan & Rajcan, 2002), India (Bhartiya et al., 2017; Samyuktha et al., 2020), northwestern Ethiopia (Atnaf et al., 2013), and South Africa (Mwiinga et al., 2020). These regions have employed the method to investigate the grain yield stability of diverse soybean cultivars across different environmental conditions, aiming to introduce appropriate cultivars for various habitats. Given the increasing need for stable soybean cultivars with optimal performance, soybean breeders are encouraged to focus on developing high‐yielding cultivars that also exhibit high stability.

Evaluating genotypes using sets of traits enhances the probability of identifying the ideal genotype. The Selection Index of Ideal Genotype (SIIG) is a multivariate statistical method that identifies the ideal genotype based on a sum of traits or various indices (Zali et al., 2023). It is noteworthy that as the number of traits or indicators increases, selecting the right genotype can become challenging. However, with the help of the SIIG discriminator, all parameters, despite having different measurement units, are consolidated into one parameter, simplifying the ranking and determination of the superior genotype (Najafi Mirak et al., 2018). This approach has been applied to a few crops, including bread wheat (Yaghutipoor et al., 2017), durum wheat (Najafi Mirak et al., 2018; Ramzi et al., 2018), lentil (Amiri et al., 2021), rapeseed (Abdollahi Hesar et al., 2021), sugar beet (Taleghani et al., 2022), and barley (Zali et al., 2023), for the assessment of superior genotypes according to a set of attributes or scales. However, the SIIG method has not yet been utilized in soybean breeding for selecting genotypes based on different traits. Therefore, the aims of this research were to (1) investigate genotype (G), environment (E), and genotype–environment (GE) interactions on the seed yield of soybean pure lines; (2) identify the most stable lines in terms of yield and stability; (3) study the relationship among environments; and (4) select superior soybean line(s) based on the SIIG index.

## 2 MATERIALS AND METHODS

### 2.1 Plant material and field

Thirteen new soybean lines, along with control genotypes (Saba and Amir), were evaluated using a randomized complete block design with three replications across four locations: Karaj, Mazandaran, Golestan, and Moghan, during two growing seasons (2020–2021 and 2021–2022). Additional details about the locations and soybean lines studied are provided in Tables 1 and 2. Each experimental plot consisted of four rows, each 5 meters in length, with a spacing of 50 cm between rows and 6 cm between plants within a row. All agronomic operations, including thinning, weed, pest, and disease control, were carried out during the growth period of the soybean plants according to the cultivation instructions specific to each zone. Phenological parameters such as days to flowering and days to maturity were recorded during the growth period of the soybeans. At maturity, five plants were selected from each plot for the measurement of agronomic properties, including plant height, node number per plant, number of pods per branch, total pod number, plant weight, number of seeds per plant, seed weight per plant, and seed yield.

**TABLE 1:** Agro‐climatic characteristics of the environments studied in this research.

Location	Latitude (N)	Longitude (E)	Elevation (m.a.s.l)	Average temperature (°C)	Rainfall (mm)	Soil type
Karaj	35.8439°	50.9715°	1302	14.1	41.71	Sandy loam
Mazandaran	36.22	52.53	989 m	17.6	53.2	Sandy loam
Golestan	37.28	55.13	38.78	21.1	30.59	Sandy clay loam
Moghan	38.25	48.29	1353	21.5	80.7	Clay loam

Abbreviations: m.a.s.l, meter above sea level; mm, millimeter; the average temperature is for two years of experiment.

**TABLE 2:** The name and pedigree of each soybean line.

Number	Code	Maternal	Paternal
1	SOY‐98‐1	Hamilton	Karbin
2	SOY‐98‐2	Hamilton	Karbin
3	SOY‐98‐6	Valenta	Karbin
4	SOY‐98‐7	Valenta	Karbin
5	SOY‐98‐11	Hamilton	TMS
6	SOY‐98‐15	Hamilton	Sari
7	SOY‐98‐16	Hamilton	Gorgan 3
8	SOY‐98‐17	Telar	Williams
9	SOY‐98‐18	Sari	Charleston
10	SOY‐98‐19	Sari	Charleston
11	SOY‐98‐20	Sari	Charleston
12	SOY‐98‐22	Williams	Clary
13	SOY‐98‐23	Sahar	Sari
14	Saba		
15	Amir		

### 2.2 Statistical analysis

The combined analysis of variance was conducted to determine the effects of genotype, environment, and genotype–environment (G × E) interaction, assuming “year” and “location” were random factors in all environments, using SAS version 9.4 (Vargas et al., 2013). To evaluate the stability of the seed yield of soybean lines, various univariate statistics were employed, including the coefficient of determination (R2), regression coefficient, and variance deviation from regression (Eberhart & Russell, 1966), along with stability variance (Shukla, 1972), Wricke's ecovalence (Wricke, 1962), and the stability coefficient (Lin & Binns, 1988), analyzed by R‐META (Pour‐Aboughadareh et al., 2019). For a better assessment of the interaction effect of genotype–environment and determination of the ideal genotype, both AMMI and GGE biplot methods were utilized using Genstat (version 12) to graphically represent the stable soybean lines (Yan et al., 2000). Finally, the principal component analysis (PCA) and the selection index of ideal genotype (SIIG) methods were applied using STATGRAPHICS (version 18) (Adilova et al., 2020) and Excel (Mau et al., 2019) to select the superior soybean line.

## 3 RESULTS

### 3.1 Analysis of variance and mean comparison of each trait

The combined analysis of variance (ANOVA) showed that all studied traits were significantly affected by the main effects of genotype, environment, and genotype–environment (G × E) interaction (Table 3). Table 4 revealed that the average plant height was 88.42 cm, with the highest and lowest heights being 115.1 cm for line 8 and 64.9 cm for line 2, respectively. The node number ranged between 12.8 (line 2) and 18.6 (line 1), averaging 15.62. The maximum and minimum branch numbers were observed in soybean lines 8 (3.5) and 3 (1.9), with an overall average of 2.72. The average number of total pods was 65.94, ranging from 50.2 (line 10) to 80.4 (line 2). The heaviest and lightest plant weights were recorded for line 12 (87.7) and line 10 (48.7), respectively. The highest seed yields belonged to line 2 (3348.7 kg ha^−1^) and line 5 (3142.8 kg ha^−1^). The earliest maturing lines were lines 1 and 5, both reaching maturity in 128 days. Taking into account seed performance and maturity, line 5 was ranked as the top‐performing line among the others (Table 4).

**TABLE 3:** Combined analysis of variance different traits of 15 soybean lines across 8 environments (combination of years and locations).

Source	Df	Days to flowering (R1)	Days to maturity (R8)	Plant height (cm)	Node number per plant	Number of branches per plant	Total pod number	Plant weight (g)	Number of seeds per plant	Seed weight per plant	Seed yield (kg ha^−1^)
Environment	3	6335.9**	3437.0**	3292.5**	672.2**	38.2**	11510.5**	10546.6**	236303.3**	13298.2**	50279213.1**
Genotype	14	938.7**	2301.7**	5088.8**	71.9**	8.7**	1910.4**	2137.5**	9335.6**	246.0**	766058.6**
Genotype × environment	42	198.4**	391.4**	660.3**	15.8**	3.9**	1894.7**	2688.0**	10942.4**	231.6**	1088143.4**
Error	224	1.13	1.84	74.89	1.86	0.52	106.24	104.81	468.21	10.12	12.90
Coefficient of variance		1.94	0.97	9.78	8.71	26.6	15.6	15.62	14.80	13.34	12.90

** Significant at *p* ≤ .01.

**TABLE 4:** The mean comparison of different traits in 15 soybean lines across 8 environments (combination of years and locations).

Number	Soybean line code	Days to flowering (R1)	Days to maturity (R8)	Plant height (cm)	Node no.	Branch no.	Total pods	Plant weight (g)	Total seeds per plant	Yield per plant	Seed yield (kg ha^−1^)
1	SOY‐98‐1	48.5^h^	128.3^m^	108.3^b^	18.6^a^	2.8^cde^	74.6^abc^	70.6^bc^	161.4^bc^	25.1^bc^	2764.5^ef^
2	SOY‐98‐2	56.1^f^	140.1^i^	64.9^j^	12.8^f^	2.6^def^	80.4^a^	55.6^e^	173.7^ab^	28.5^a^	3348.7^a^
3	SOY‐98‐6	49.2^g^	129.2^L^	99.6^cd^	17.1^b^	1.9^h^	55.5^gh^	70.8^bc^	130.6^fg^	21.4^e^	2774.2^def^
4	SOY‐98‐7	47.7^i^	129.9^kl^	103.6^bc^	16.7^bc^	2.0^h^	54.3^gh^	64.5^d^	122.4^gh^	20.8^e^	2879.4^cde^
5	SOY‐98‐11	45.1^j^	128.3^m^	77.4^hi^	16.3^bc^	2.1^gh^	74.5^abc^	68.2^cd^	178.7^a^	27.0^a^	3142.8^ab^
6	SOY‐98‐15	57.3^e^	145.1^f^	84.3^g^	16.1^c^	2.5e^fg^	63.0^f^	69.3^bcd^	145.8^de^	24.3^c^	2992.5^bc^
7	SOY‐98‐16	59.3^d^	147.4^e^	82.0^gh^	14.7^d^	3.2^bc^	56.7^g^	66.7^cd^	131.1^fg^	23.9^cd^	2822.1^cde^
8	SOY‐98‐17	57.0^e^	148.3^d^	115.1^a^	18.5^a^	3.5^b^	66.6^def^	67.5^cd^	167.3^abc^	27.2^a^	2995.0^bc^
9	SOY‐98‐18	55.6^f^	141.9^h^	68.0^j^	13.7^e^	2.6^def^	65.7^ef^	57.1^e^	126.4^fgh^	18.4^f^	2901.3^cde^
10	SOY‐98‐19	59.4^d^	143.4^g^	74.6^j^	13.7^e^	2.4^efg^	50.2^h^	48.7^f^	115.9^h^	18.9^f^	2959.9^bcde^
11	SOY‐98‐20	47.9^i^	130.9^j^	92.8^ef^	16.8^bc^	2.3^fgh^	71.7^bcd^	63.7^d^	137.4^ef^	21.1^e^	2840.9^cde^
12	SOY‐98‐22	64.2^a^	154.9^a^	97.4^de^	14.9^d^	4.2^a^	66.0^def^	87.7^a^	144.6^de^	27.0^a^	2590.4^f^
13	SOY‐98‐23	61.4^c^	153.6^b^	81.5^gh^	14.7^d^	3.1^bc^	77.0^ab^	63.6^d^	155.4^cd^	25.0^bc^	2787.3^cdef^
14	Saba	48.3^hi^	130.0^k^	91.4^f^	15.0^d^	2.6^def^	69.1^cde^	74.8^b^	164.8^bc^	26.8^ab^	2980.5^bcd^
15	Amir	63.2^b^	149.7^c^	85.5^g^	14.7^d^	3.0^cd^	63.9^ef^	54.2^ef^	136.3^ef^	22.2^de^	2817.3^cde^
	Total mean	54.68	140.02	88.42	15.62	2.72	65.94	65.53	146.12	23.84	2906.45

*Note*: The superscript letters in each column explain no significant.

### 3.2 Additive main effects and multiplicative interaction (AMMI) analysis for grain yield

The AMMI analysis for seed yield demonstrated a highly significant effect of environment, genotype, and genotype–environment interaction. The effects were rated as E > GEI > G, contributing 65.89%, 29.58%, and 4.5%, respectively (Table 5). The AMMI model, based on partitioning the GEI, indicated that the first five terms of AMMI were significant, explaining 96.35% of the GEI variance. Specifically, the first and second principal component axes (IPCA) of the interaction explained 48.70% and 24.97% of the GEI sum of squares, respectively (Table 5). Among the eight testing environments, seed yields were highest in Moghan in the first year, with an average of 4118 kg ha^−1^, followed by Moghan in the second year with 3892 kg ha^−1^ (Table 6). Conversely, the lowest seed yields were recorded in Mazandaran in the second year (2342 kg ha^−1^) and the first year (2346 kg ha^−1^). The mean grain yield of genotypes across environments (Table 6) showed that line 2 (3348.7 kg ha^−1^) and line 5 (3142 kg ha^−1^) were the highest yielding genotypes, while genotype line 12 yielded the lowest at 2590.4 kg ha^−1^.

**TABLE 5:** Additive main effect and multiplicative interaction analysis of variance for seed yield (kg ha^−1^) of soybean genotypes across eight environments.

Source	Df	Sum of square	Mean square	Treatments (%)	Genotype × environment interaction (%)
Total	359	271,142,356	755,271		
Treatments	119	237,432,904	1,995,234**		
Genotypes	14	10,724,953	766,068**	4.51	
Environments	7	156,459,204	22,351,315**	65.89	
Block	16	2,210,413	138,151^ns^		
Genotype × environment Interactions	98	70,248,748	716,824**	29.58	
IPCA1	20	34,213,697	1,710,685**		48.70
IPCA2	18	17,541,359	974,520**		24.97
IPCA3	16	8,050,801	503,175**		11.46
IPCA4	14	4,493,691	320978**		6.39
IPCA5	12	3,325,199	277,100*		4.73
IPCA6	10	1,748,336	174,834		2.48
IPCA7	8	875,664	109,458		1.24
Error	224	31,499,039	140,621		

Abbreviations: IPCA 1 and IPCA 2, interaction principal component axis one and two, respectively.

* Significant at .05 probability level;

** Significant at *p* ≤ .01.

**TABLE 6:** Mean grain yield (kg ha^−1^) and environment and genotype IPCA1 scores for 15 genotypes tested at four environments during 2020–21 and 2021–22.

Code	Genotypes	Environments								Genotype mean	IPCA1
		Karaj 1	Moghan 1	Mazandaran 1	Golestan 1	Karaj 2	Moghan 2	Mazandaran 2	Golestan 2		
1	SOY‐98‐1	1319.2	3651.5	2936.0	2313.3	3217.8	3955.0	2647.8	2075.3	2764.5	−18.72
2	SOY‐98‐2	2762.9	4765.2	3091.3	2791.0	3515.7	3876.8	3314.1	2672.3	3348.7	−14.95
3	SOY‐98‐6	2180.1	3280.9	1983.7	2774.3	2953.8	3881.1	2136.4	3003.0	2774.2	8.94
4	SOY‐98‐7	2512.9	3865.9	1555.0	3422.0	2901.2	3976.1	1431.9	3370.3	2879.4	26.57
5	SOY‐98‐11	2782.0	4375.6	2111.0	3312.0	3302.2	4391.6	2483.8	2384.3	3142.8	5.15
6	SOY‐98‐15	3505.3	4071.1	2530.0	2303.7	3239.7	3292.4	2594.7	2403.0	2992.5	−3.96
7	SOY‐98‐16	3409.1	4551.9	2314.7	2303.7	2443.3	3642.2	1597.3	2314.3	2822.1	5.33
8	SOY‐98‐17	1862.6	4842.5	3583.0	1976.7	2307.0	3808.3	3758.7	1821.0	2995.0	−40.07
9	SOY‐98‐18	2727.0	4238.5	2144.7	3303.3	2539.9	3756.2	1735.4	2765.7	2901.3	13.39
10	SOY‐98‐19	2338.1	4470.0	2215.7	3300.0	2772.3	3838.7	1999.2	2745.3	2959.9	7.66
11	SOY‐98‐20	2086.6	3532.2	2088.7	3098.3	2801.7	4254.7	2400.3	2464.7	2840.9	3.71
12	SOY‐98‐22	1134.9	4345.9	2068.0	2419.3	2628.3	3502.1	2061.0	2563.7	2590.4	−5.64
13	SOY‐98‐23	2174.4	4024.4	2148.3	2978.0	2084.7	3609.6	2316.1	2963.0	2787.3	4.23
14	Saba	2806.7	4310.4	2210.0	2964.7	2252.4	4262.3	2309.5	2728.3	2980.5	5.61
15	Amir	1935.1	3449.4	2214.7	2713.3	2645.8	4332.9	2352.4	2895.0	2817.3	2.73
Mean		2369.1	4118.4	2346.3	2798.2	2773.7	3892.0	2342.6	2611.3	2906.5	
Env. IPCA1		17.09	−9.83	−28.35	24.63	0.72	5.62	−32.30	22.42		

Abbreviations: Env., environment; IPCA 1, interaction principal component axis.

### 3.3 Stability analysis

#### 3.3.1 Evaluation of various parametric stability

The yield stability criteria were evaluated using various parametric methods (Table 7). The data indicated that the mean square of each line was significant for seed yield. According to the regression coefficient, lines 5, 8, 10, 12, and 14 were more sensitive to environmental changes and had specific adaptability to high‐efficiency environments, as indicated by their regression coefficients being greater than 1. Conversely, lines 1, 2, 3, and 6 exhibited the highest tolerance to environmental changes and adaptability to low‐efficiency environments due to regression coefficients less than 1.

**TABLE 7:** The parametric stability criteria in the studied soybean lines.

Line no.	Soybean line code	Mean	Wricke's ecovalence	Shukla's stability variance	Regression coefficient	Lin and Binns	BLUP	*R* ^2^	Coefficient of variation (CV)	Deviation from regression parameter
1	SOY‐98‐1	2764	2323684.5	361811.9	0.92	367,869	2897.3	.56	31.2	383241.3
2	SOY‐98‐2	3349	905217.9	133427.1	0.90	71,566	2934.9	.75	21.1	145021.9
3	SOY‐98‐6	2774	1482036.8	228507.1	0.73	350,518	2898.0	.58	23.4	205894.9
4	SOY‐98‐7	2879	3763896.0	604637.8	0.95	400,518	2904.7	.44	34.0	625907.0
5	SOY‐98‐11	3143	1376711.6	211145.8	1.11	185,440	2921.6	.75	27.8	223251.3
6	SOY‐98‐15	2993	2436651.5	385861.2	0.55	217,645	2912.0	.36	21.0	296599.6
7	SOY‐98‐16	2822	2602507.9	413200.2	1.09	348,385	2901.0	.60	34.0	429596.4
8	SOY‐98‐17	2995	4352392.5	701642.7	1.24	299,634	2912.1	.55	38.2	694394.7
9	SOY‐98‐18	2901	1478381.0	227904.5	1.01	295,622	2906.1	.69	28.5	246365.4
10	SOY‐98‐19	2960	1006003.4	150040.1	1.13	257,681	2909.9	.81	28.9	158588.2
11	SOY‐98‐20	2841	1023582.4	152937.7	0.96	308,719	2902.2	.75	26.7	169651.3
12	SOY‐98‐22	2590	1395231.5	214198.5	1.30	475,936	2886.1	.83	37.5	183639.4
13	SOY‐98‐23	2787	883326.8	129818.6	0.94	320,077	2898.8	.77	26.3	145439.1
14	Saba	2981	950057.8	140818.3	1.13	233,022	2911.2	.82	28.6	148770.1
15	Amir	2817	1045643.9	156574.2	0.97	324,818	2900.7	.75	27.2	173716.0
Maximum	–	3349	4,352,393	701642.7	1.30	475,936	2934.9	.83	38.2	694394.7
Minimum	–	2590	883326.8	129818.6	0.55	71,566	2886.1	.36	21.0	145021.9

Regarding the Shukla method, lower Shukla stability variance corresponded to greater genotype stability. Consequently, lines 2, 10, 13, and 14 showed lower Shukla stability variance, indicating high stability, whereas lines 4, 6, 7, and 8 exhibited low stability. According to Wricke's ecovalence stability parameter, lines 2, 10, 13, and 14 had the lowest coefficients, marking them as the most stable lines, while lines 8, 4, 7, and 6, with the highest ecovalence, were considered the least stable.

The mean deviation from regression is another stability factor, with genotypes closer to zero or having minimal deviation being more stable. Therefore, lines 2, 10, 13, and 14 were the most stable, having the least deviation from regression. Lines 1, 4, 7, and 8 exhibited the highest deviation from regression, indicating lower stability. The coefficient of determination (R2) data showed that lines 4 and 6, with the lowest R2, were more stable. The coefficient of variation (CV) results indicated that genotypes with lower CV, such as lines 2 and 6, had higher stability. According to the Lin and Binns indicator, lower values of this parameter signify greater stability. Hence, lines 2, 5, 6, and 14 were selected as the most stable lines. Furthermore, the BLUP stability measurement, an effective approach for multi‐environmental analysis, was used to estimate genotype means with high accuracy, reducing G × E interaction, and selecting high‐yield genotypes based on a combination of stability and performance. Based on this parameter, lines 2, 5, and 8 were identified as the most stable lines (Table 7).

#### 3.3.2 Polygon view of GGE biplot to group lines and environment

**FIGURE 1:**
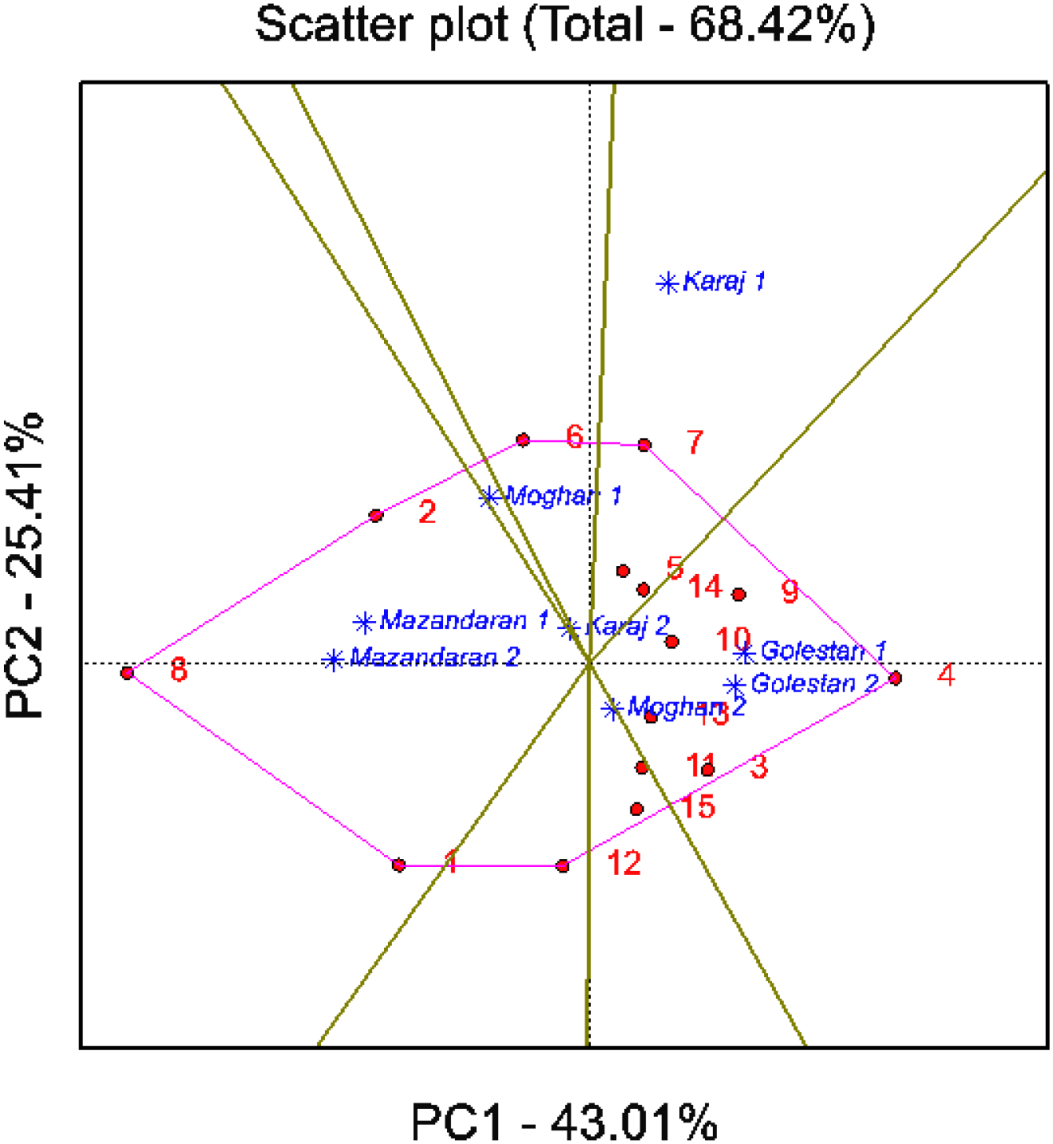
The polygon view of the GGE biplot to show mega‐environments and their superior lines. Refer to Tables 1 for line name.

In the polygon view, the soybean lines positioned at each vertex of a sector indicated the best‐performing lines in the test environment corresponding to that particular sector, identified by the greatest distance from the center. According to the vertical lines of the polygon side, there were five mega‐environments (MEs) encompassing six soybean lines (Figure 1). The first ME included two out of eight sectors (Golestan 1 and 2) with line 4 being superior and showing the highest yield of 2879 kg ha^−1^. The second ME comprised Moghan 2, where soybean line 15 had the highest yield of 2817 kg ha^−1^. The third ME consisted of two sectors, Mazandaran 1 and 2, with superior soybean lines 2, 8, and 1 yielding 3348, 2995, and 2764 kg ha^−1^, respectively. The fourth ME included Karaj 2 and Moghan 1, with soybean line 6 yielding 2992 kg ha^−1^ (Figure 1). The last ME contained Karaj 1, where soybean line 7 yielded 2822 kg ha^−1^. Soybean line 12 was not considered superior and showed the least yield in all MEs. Furthermore, lines 5, 10, 13, and 14, with yields of 3142, 2959, 2787, and 2980 kg ha^−1^, respectively, exhibited the least genotype–environment (GE) interaction, emphasizing their suitable general adaptability to all environments (Figure 1).

#### 3.3.3 GGE biplot analysis based on comparison of all lines with ideal line

**FIGURE 2:**
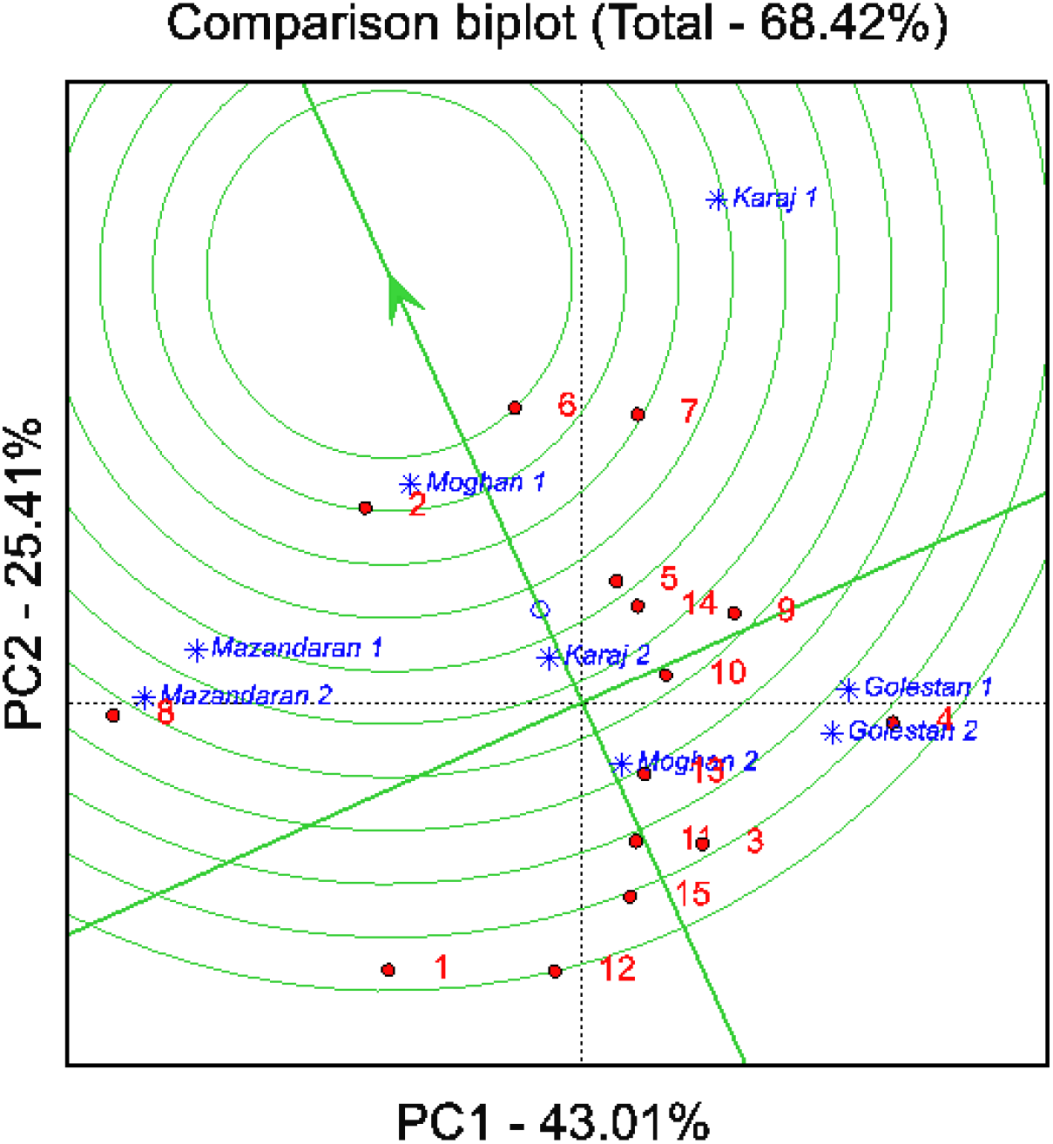
Biplot view to compare the studied lines with the ideal line. Refer to Tables 1 for line name.

The ideal genotype should exhibit the highest average performance and stability across the tested environments. Such a genotype is represented by the longest vector length among genotypes with high average performance, coupled with a minimal contribution to genotype–environment (GE) interaction. Although an ideal genotype in this precise form may not exist in practice, it serves as a benchmark for genotype evaluation. The proximity of a genotype to this reference indicates its ideality. For the assessment of the ideal genotype, GGE biplots use concentric circles to graphically determine the distance between the studied genotype and the ideal genotype (Figure 2). Consequently, soybean lines 6, 2, 7, and 5, with yields of 2992, 3348, 2822, and 3142 kg ha^−1^, respectively, were selected as ideal genotypes due to their proximity to the ideal genotype and their high yield and stability (Figure 2). In contrast, lines 12, 1, 15, and 4, with seed yields of 2590, 2764, 2817, and 2879 kg ha^−1^, respectively, were identified as less favorable genotypes based on their greater distance from the ideal genotype (Figure 2). It is important to note that examining stability alone is not sufficient; less stable cultivars with good average performance can be preferable over stable cultivars with inadequate yields.

#### 3.3.4 Biplot view for synchronous evaluation of lines based on stability and yield

**FIGURE 3:**
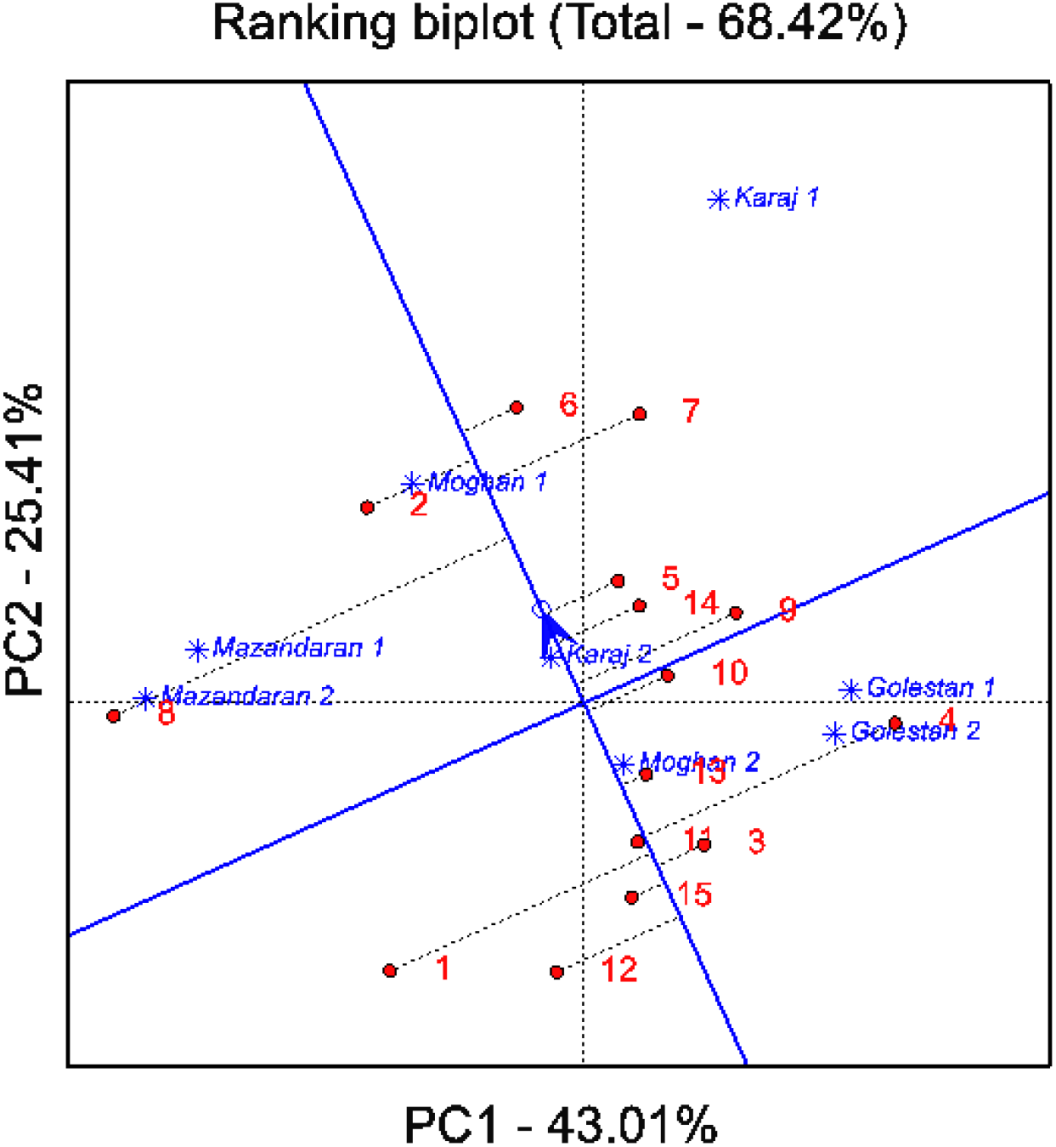
Biplot view for simultaneous selection of seed yield and stability of the studied lines. Refer to Tables 1 for lines name.

An important aspect of the GGE biplot model is the simultaneous assessment of genotypes according to yield and stability. Figure 3 displays the ranking of 15 soybean lines according to their seed yield and stability across various environments.

The genotype is positioned into nearness to the concentric rings, determining the best‐performing genotype, and the projection from the average environment axes (AEA) abscissa indicates the genotype's stability. Genotypes are considered being more stable when they are placed on the horizontal axis (AEC abscissa) and have zero projection from the vertical axis (AEC ordinate), while the genotype with the longest direction from the AEC abscissa is treated as unstable; a similar report was stated by Oladosu et al. (2017).

The line marked with an arrow, representing the average coefficients of the first two components of the GGE biplot model, passes from the center of the biplot to the desired point and is known as the average environment coordinate (AEC). Genotypes closer to the concentric rings demonstrate the best‐performing genotypes and the projection from average environment axes (AEA) abscissa indicates the genotype's stability. The more distance of genotypes from AEC, the less stable genotypes appear (Oladosu et al., 2017). According to the results, soybean lines 15, 11, 13, and 3 exhibited the highest stability with average yields of 2817, 2840, 2787, and 2774 kg ha^−1^, respectively (Figure 3). Lines 6, 5, and 2 showed average stability with yields of 2992, 3142, and 3348 kg ha^−1^, respectively. Conversely, lines 8, 1, and 4 were more unstable with performances of 2995, 2764, and 2879 kg ha^−1^, respectively (Figure 3).

Additionally, these lines were grouped into clusters along the vertical line on the AEC. The first group (upper) and the second group (lower) contained lines with higher and lower average yields than the median yield. Consequently, lines 6 and 5 were identified as the most stable lines with yields of 2992 and 3142 kg ha^−1^, respectively (Figure 3).

### 3.4 Selection index of ideal genotype (SIIG)

In this study, the selection index of ideal genotype (SIIG) was calculated based on 11 traits, excluding seed yield, to select the best genotypes. The ideal genotype was defined as the one with higher values in traits such as plant height, node number per plant, number of branches per plant, number of pods per branch, total pod number, plant weight, number of seeds per plant, and seed weight per plant, and lower values in days to flowering, days to pod formation, and days to maturity. By utilizing the SIIG index, these attributes are consolidated into a single index, enabling more reliable and accurate selection of superior genotypes. The SIIG index ranges between 0 and 1. A genotype with an SIIG close to 1 indicates a more favorable condition in terms of most investigated traits, while a lower SIIG suggests less desirable conditions. According to the data, soybean lines 8, 1, and 5 emerged as superior with the highest SIIG values of 0.687, 0.651, and 0.634, respectively (Table 8). Conversely, lines 10, 3, and 15 had the lowest SIIG values of 0.215, 0.387, and 0.405, respectively.

**TABLE 8:** The SIIG of soybean lines according to all traits studied following distance from desirable line (d^+^), none desirable line (d^−^), and seed yield.

Number	Genotype name	d^+^	d^−^	SIIG	Seed yield
1	SOY‐98‐1	0.155	0.289	0.651	2764
2	SOY‐98‐2	0.196	0.272	0.582	3349
3	SOY‐98‐6	0.294	0.186	0.387	2774
4	SOY‐98‐7	0.274	0.190	0.409	2879
5	SOY‐98‐11	0.154	0.267	0.634	3143
6	SOY‐98‐15	0.181	0.212	0.540	2993
7	SOY‐98‐16	0.225	0.212	0.486	2822
8	SOY‐98‐17	0.166	0.365	0.687	2995
9	SOY‐98‐18	0.233	0.234	0.501	2901
10	SOY‐98‐19	0.326	0.089	0.215	2960
11	SOY‐98‐20	0.250	0.194	0.437	2841
12	SOY‐98‐22	0.219	0.359	0.621	2590
13	SOY‐98‐23	0.177	0.285	0.617	2787
14	Saba	0.184	0.245	0.571	2981
15	Amir	0.256	0.174	0.405	2817

**FIGURE 4:**
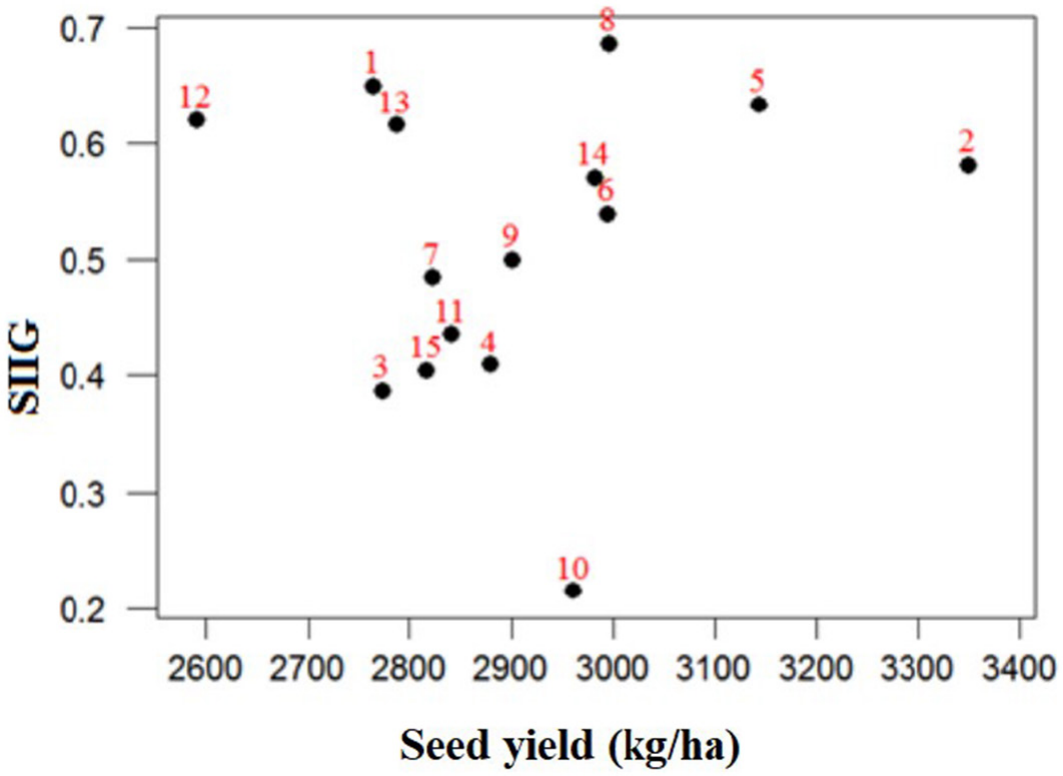
The two‐dimensional of distribution of 15 soybean lines based on seed yield and SIIG.

Additionally, a two‐dimensional graph was used for the simultaneous selection of lines based on agronomic traits and seed yield. Figure 4 showed that soybean lines 1, 5, and 8 were superior, having higher SIIG and seed yield. However, lines 3 and 15 were identified as the least desirable lines in terms of the traits studied.

### 3.5 Principal component analysis (PCA)

The results indicated that the first three components, each with values higher than 1, accounted for 81.22% of the total variance of variables (Table 9). In the first principal component (PC1), the phenological traits included days to flowering (DAF), days to pod formation (DAPF), and days to maturity (DAM) with coefficients of 0.46, 0.47, and 0.46, respectively. In PC2, the highest coefficients were associated with the total number of pods (0.47), seed weight per plant (0.49), and the number of seeds per plant (0.54). Finally, for PC3, the parameters of plant weight, node number, and plant height showed the highest positive coefficients at 0.45, 0.49, and 0.57, respectively, while seed yield had the highest negative coefficient (−0.44).

**TABLE 9:** The results of principal component analysis for all traits studied.

Traits	Component 1	Component 2	Component 3
Days to flowering (DAF) (R1)	0.46	−0.16	0.08
Days to pod formation (DAPF) (R3)	0.47	−0.11	0.06
Days to maturity (DAM) (R8)	0.46	−0.09	0.03
Plant height (PH)	−0.09	0.02	0.57
Node number per plant (NN/P)	−0.22	0.07	0.49
Number of branches per plant (NB/P)	0.40	0.19	0.14
Number of pods per branch (NP/B)	0.33	0.25	−0.08
Total pod number (TP)	0.06	0.47	−0.10
Plant weight (PW)	−0.03	0.24	0.45
Number of seeds per plant (NS/P)	−0.02	0.54	−0.02
Seed weight per plant (SW/P)	0.09	0.49	0.02
Seed yield (Y)	−0.15	0.21	−0.44
Specific variance	3.94	3.22	2.58
Percentage of variance	32.87	26.81	21.54
Percentage of cumulative of variance	32.87	59.68	81.22

**FIGURE 5:**
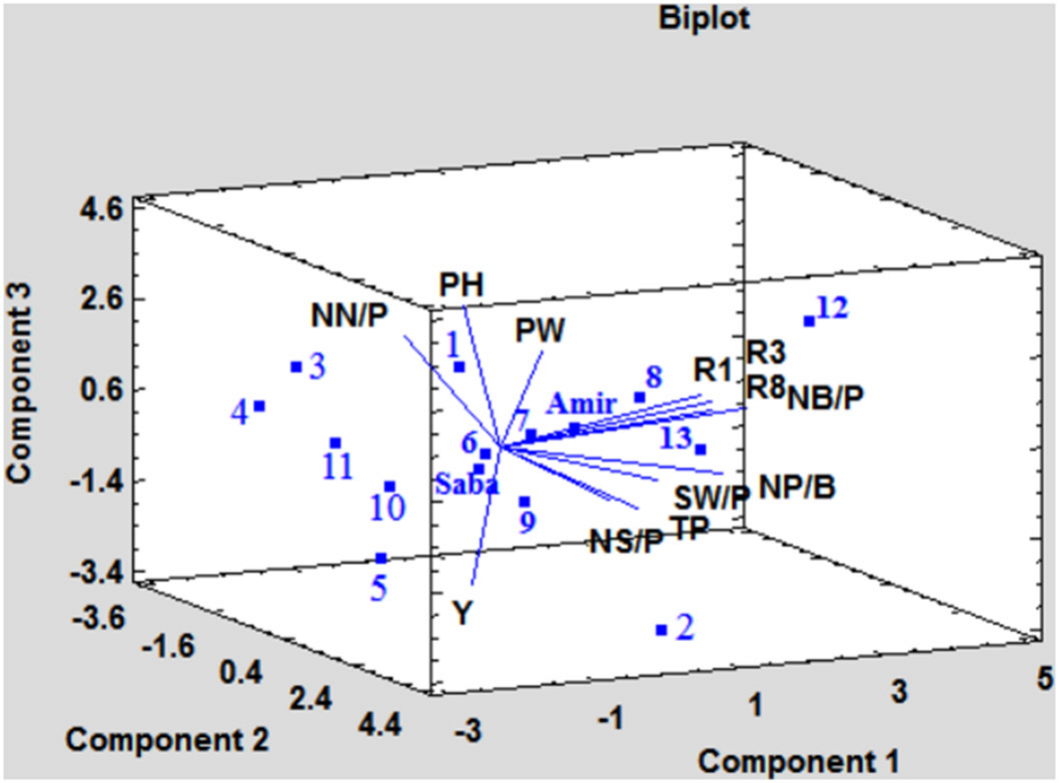
The three significant components plot obtained by principal component analysis in order to locate traits and lines. Y: yield; NN/P: number of nodes per plant; PH: plant height; PW: plant weight; R1: days to flowering (DAF); R2: days to pod formation (DAPF); R3: days to maturity (DAM); NB/P: number of branches per plant; NP/B: number of pods per plant; SW/P: seed weight per plant; NS/P: number of seeds per plant; TP: total pods per plant.

Figure 5 displays the location of each trait and genotype on a plot generated using PC1, PC2, and PC3. The graphs for traits that are related to each other align in the same direction, whereas those with inverse relationships do not align. This figure enables the selection of different lines based on various traits. A line is drawn from the related trait to the origin of the coordinate system, followed by another line passing through the origin, which is vertical to the first line. Lines with the highest projection on the first line and located on the side of the concerned trait relative to the vertical line have the maximum value of that trait. Conversely, the minimum values of the traits are determined by the direction opposite to the concerned traits relative to the vertical line. For instance, lines 2 and 5, with the highest positive projections for the seed yield parameter, could be selected as high‐yield lines (Figure 5). Additionally, lines 12 and 8, with the highest projections but in the opposite direction to seed yield, are indicated as lines with low yield. Line 5 was also selected for its high yield and earliness (Figure 5).

## 4 DISCUSSION

A significant portion of Iran's annual income is allocated to importing oilseeds like soybeans for essential oils and proteins needed in human and livestock diets. Consequently, increasing soybean cultivation area and yield is crucial for reducing reliance on imports. Soybean in Iran has adapted to various environmental conditions, making the development of genotypes with high adaptability to diverse environments a key goal in plant breeding.

The performance stability of a variety refers to its consistent production in a specific environment over time (Mirza et al., 2013). A plant variety's ability to tolerate different stresses (such as temperature extremes, water levels, day length changes, and light intensity) and survive a range of soil conditions is essential for growth and development (Kumaresan & Nadarajan, 2010). Genotype and environment interaction (G × E interaction) occurs when plant genotypes respond differently to environmental changes, impacting the relationship between phenotypic and genotypic values. This interaction reduces the effectiveness of the selection process in plant breeding, which relies heavily on accurate phenotypic predictions (Dia et al., 2016). Addressing this requires selecting stable genotypes with minimal G × E interaction and developing genotypes with lower G × E interaction effects by dividing heterogeneous environments into more homogeneous ones (Farshadfar et al., 2011).

In the present study, the stability of 15 soybean lines under various Iranian environmental conditions were evaluated. Combined ANOVA analysis revealed that genotype, environment, year, and their interactions significantly influenced all traits studied. The environment contributed 65.89% to yield variability, more than genotypes (4.5%) and G × E interaction (29.58%), indicating considerable environmental variability across different climatic conditions. This finding aligns with reports by Liu et al. (2017), Hailemariam Habtegebriel (2022), and Susanto et al. (2023), underscoring the significant influence of G × E interaction on soybean yield, which reflects the varied responses of genotypes to different environments.

When the presence of GEIs was confirmed through combined ANOVA analysis and the AMMI model, various stability measurements were used to evaluate the stability and compatibility of genotypes across different environments. Researchers have employed several methods for multi‐environmental examination to accurately select stable and high‐yielding lines, as referenced by Ahmadi et al. (2015), Vaezi et al. (2019), and Maulana et al. (2020). Table 4 in the study delineates different stability measurements that are used to select various stable lines, each based on different assumptions about calculating stability and adaptability.

The stability parameters of Wrick ecovalence and Shukla variance demonstrated similar results, confirming the affinity between these parameters in determining stable genotypes, as reported by Shojaei et al. (2021) in maize hybrids. Prior research has shown that the Wrick and Shukla stability methods identified certain barley genotypes, like Centeno and ICNBF 8–611, as highly stable, while others such as Gloria and Ehbytm80‐1 were noted as low stable genotypes (Bahrami et al., 2009). Our findings highlighted that soybean lines 2 and 5, with yields of 3349 and 3143 kg ha^−1^, respectively, were selected as the more stable lines among others. This was based on stability factors such as lower values of *Wi* ^*2*^, *σ* ^*2i*^, *P* ^*i*^, and *s* ^*2*^ *di*, a high BLUP factor, and bi equal to 1. The use of different parametric stability assessments has also been reported in wheat (Bornhofen et al., 2017), rice (Lee et al., 2023), safflower (Afzal et al., 2021), and oat (Kebede et al., 2023).

Based on multivariate analytical methods, the AMMI and GGE biplot are valuable tools for identifying stable lines in various environments. These tools consider different characteristics related to the performance of the tested lines (Zhang et al., 2016). Ruswandi et al. (2021) have highlighted the effectiveness of the GGE biplot in graphically displaying the relationships between genotype, environment, and G × E interactions. The GGE biplot's polygon view, known as “which won where”, is a critical graphical pattern for recognizing mega environments and determining the best genotype for each. This approach has been applied in numerous studies across different plant species, including groundnuts. In the current study, the polygon plot of the GGE biplot revealed five mega environments, each with specific superior lines identified as vertex lines (1, 2, 4, 6, 7, 8, 12). These lines performed better or worse in some or all environments due to their distance from the biplot's origin. Conversely, soybean lines 5, 10, 13, and 14 were near the biplot origin, indicating average performance and lower GEI variation compared to the vertex genotypes. This pattern aligns with findings from Adham et al. (2022), Bilate Daemo et al. (2023), Esan et al. (2023), and Kindie et al. (2022), who reported on dividing testing environments into various mega environments with different sectors and numbers of genotypes.

The GGE biplot has the unique capability of identifying the highest yielding genotypes across different environments, referred to as the “ideal genotype”. This tool is particularly effective due to its ability to predict both the average yield of a genotype and its stability and adaptability to specific environments, as outlined by Santos et al. (2017). In our study, soybean lines 2, 5, 6, and 7 were identified as desirable due to their proximity to the ideal genotype. This finding aligns with the work of Ansarifard et al. (2020) and Stansluos et al. (2023), who employed the GGE biplot for evaluating and selecting outstanding genotypes based on various agronomic traits. While the outcomes of parametric stability analysis methods largely concurred with the GGE biplot analysis, additional stability approaches were necessary to categorize stable lines and reinforce our results. The findings of our study are in agreement with previous research conducted by Mohammadi and Amri (2008) and Ruswandi et al. (2022).

Another tool for ranking and comparing various genotypes is the SIIG index, which is superior to other methods. The SIIG method can select the appropriate genotypes based on different traits, such as morphological and physiological characteristics. Indeed, this method can sum all traits and indexes into one index to more easily rank the best genotype (Mirzaei & Hemayati, 2021; Zali et al., 2023). According to the SIIG index in the present study, the soybean lines 8, 1, and 5 were chosen as superior lines based on the maximum amount of agronomic traits and the minimum of phenologic parameters. Zali et al. (2023) reported on the identification of superior barley genotypes using the SIIG method, which led to the introduction of genotypes 4, 8, 31, and 28 as the ideal genotypes for different tested environments. In another study, Gholizadeh et al. (2021) expressed that the SIIG index was an applicable method for effectively screening ideal genotypes in sunflowers by combining various agronomic traits.

To classify soybean lines, a multivariate method called principal component analysis (PCA) was used, which showed that the total variation exhibited by PC1, PC2, and PC3 were 32.87%, 26.81%, and 21.54%, respectively. PC1 was positively associated with phenological traits. PC2 was connected to yield components such as TP, NS/P, and SW/P. It was evident that the high coefficient of the expressed traits was due to the increment of TP and NP/B, which caused the increase of NS/P, SW/P, followed by yield (Y). PC1 was positively related to PH, NN/P, and SW/P, as well as negatively linked to Y. This issue can be explained by the fact that increasing NN/P, the plant height and subsequently plant biomass will enhance, which have a slight effect on seed yield. Indeed, when a plant becomes grassy, the seed yield decreases per surface unit. The usage of PCA has been reported in numerous studies. For instance, Girgel (2021) clustered different bean genotypes based on agronomic, morphological, and biochemical parameters using PCA. In another study, Ullah et al. (2022) reported on the utilization of PCA in cotton under drought stress, resulting in the total variation defined by PC1 (55.55%) and PC2 (41.95%). PCA has the capability of reducing and summarizing high‐dimensional data sets into smaller variables as principal components, which helps breeders when encountering too many variables (Benlioglu & Ozkan, 2022; Rencher & Christensen, 2012).

## 5 CONCLUSION

This study meticulously evaluated the adaptability of various soybean lines under diverse environmental conditions, with a specific focus on yield and stability. The findings reveal that climatic differences in the study areas significantly impacted soybean crop behavior, particularly concerning yield and its related components. Based on the results: Soybean lines 2 and 5 were chosen for their notable performance, delivering average yields of 3349 and 3142 kg ha^−1^, respectively, as per parametric factors. GGE biplot analysis highlighted soybean lines 6 and 5 as exceptionally stable, with respective yields of 2992 and 3142 kg ha^−1^. The SIIG method marked soybean lines 2, 5, and 8 as superior performers. PCA singled out soybean line 5 as the top‐ranking line, particularly for its early maturation characteristic.

In summary, this comprehensive analysis points to soybean line 5 as the most promising and adaptable option across the varied climates of Iran. The line's consistent yield and early maturity profile position it as a prime candidate for future breeding initiatives, aimed at boosting the efficiency and sustainability of soybean production.

## AUTHOR CONTRIBUTIONS

**Parastoo Majidian:** Conceptualization (equal); investigation (equal); project administration (equal); resources (equal); software (equal); supervision (equal); writing – original draft (equal); writing – review and editing (equal). **Bahram Masoudi:** Conceptualization (equal); data curation (equal); formal analysis (supporting); funding acquisition (equal); investigation (equal); methodology (equal); project administration (equal); resources (equal); software (equal); supervision (equal); validation (equal); visualization (equal). **Ebrahim Hezarjaribi:** Conceptualization (equal); investigation (equal); project administration (equal); resources (equal); supervision (equal). **Nasrin Razmi:** Investigation (equal); project administration (equal); resources (equal); supervision (equal); visualization (equal). **Kamal Peyghamzadeh:** Data curation (equal); formal analysis (equal); investigation (equal); resources (equal); supervision (equal). **Amir Gholizadeh:** Conceptualization (equal); data curation (equal); resources (equal); software (equal).

## FUNDING INFORMATION

This study was supported by grant and genetic material provision from the Seed and Plant Improvement Institute (SPII), Karaj, Iran.

## CONFLICT OF INTEREST STATEMENT

The authors declare that they have no conflict of interest.

## Data Availability

There are no data available for the statement.

## References

[fsn33996-bib-0001] Abdollahi Hesar, A. , Sofalian, O. , Alizadeh, B. , Asghari, A. , & Zali, H. (2021). Investigation of frost stress tolerance in some promising rapeseed genotypes. Journal of Agricultural Science and Sustainable Production, 31(2), 271–288. 10.22034/SAPS.2021.13109

[fsn33996-bib-0002] Adham, A. , Ghaffar, M. B. A. , Ikmal, A. M. , & Shamsudin, N. A. A. (2022). Genotype × environment interaction and stability analysis of commercial hybrid grain corn genotypes in different environments. Lifestyles, 12(11), 1773. 10.3390/life12111773 PMC9693098 36362928

[fsn33996-bib-0003] Adilova, S. S. , Qulmamatova, D. E. , Baboev, S. K. , Bozorov, T. A. , & Morgunov, A. I. (2020). Multivariate cluster and principle component analyses of selected yield traits in uzbek bread wheat cultivars. American Journal of Plant Sciences, 11(06), 903–912. 10.4236/ajps.2020.116066

[fsn33996-bib-0004] Afzal, O. , Hassan, F. U. , Ahmed, M. , Shabbir, G. , & Ahmed, S. (2021). Determination of stable safflower genotypes in variable environments by parametric and non‐parametric methods. Journal of Agriculture and Food Research, 6, 100233. 10.1016/j.jafr.2021.100233

[fsn33996-bib-0005] Ahmadi, J. , Vaezi, B. , Shaabani, A. , Khademi, K. , & Ourang, S. F. (2015). Non‐parametric measures for yield stability in grass pea (*Lathyrus sativus* L.) advanced lines in semi warm regions. Journal of Agriculture, Science and Technology, 17, 1825–1838.

[fsn33996-bib-0006] Amiri, R. , Pezeshkpour, P. , & Karami, I. (2021). Identification of lentil desirable genotypes using multivariate statistical methods and selection index of ideal genotype under rainfed conditions. Journal of Crop Breeding, 13(39), 140–151. 10.52547/jcb.13.39.140

[fsn33996-bib-0007] Ansarifard, I. , Mostafavi, K. , & Khosroshahli, M. (2020). A study on genotype–environment interaction based on GGE biplot graphical method in sunflower genotypes (*Helianthus annuus* L.). Food Science & Nutrition, 8, 3327–3334. 10.1002/fsn3.1610 32724597 PMC7382153

[fsn33996-bib-0008] Atnaf, M. , Kidane, S. , Abadi, S. , & Fisha, Z. (2013). GGE biplots to analyze soybean multi‐environment yield trial data in north Western Ethiopia. Journal of Plant Breeding and Crop Science, 5(12), 245–254. 10.5897/JPBCS13.0403

[fsn33996-bib-0009] Baghbani‐Arani, A. , Poureisa, M. , Alekajbaf, H. , Borz‐Abad, R. K. , & Khodadadi‐Dashtaki, K. (2021). Investigating the status of transgenic crops in Iran in terms of cultivation, consumption, laws and rights in comparison with the world. Scientific Reports, 11(1), 9204. 10.1038/s41598-021-88713-7 33911171 PMC8080789

[fsn33996-bib-0010] Bahrami, S. , Bihamta, M. R. , & Solouki, M. (2009). Adaptation and stability analysis of hulless barley (*Hordeum vulgare* L.) genotypes in temperate regions of Iran. Trakia Journal of Sciences, 7(2), 8–17.

[fsn33996-bib-0011] Benlioglu, B. , & Ozkan, U. (2022). Multivariate analysis and its application for screening mungbean [*Vigna radiata* (L.) wilczek] landraces. Legume Research‐An International Journal, 45(3), 299–304. 10.18805/LRF-661

[fsn33996-bib-0012] Bhartiya, A. , Aditya, J. P. , Singh, K. , Purwar, J. P. , & Agarwal, A. (2017). AMMI & GGE biplot analysis of multi environment yield trial of soybean in North Western Himalayan state Uttarakhand of India. Legume Research‐an International Journal, 40(2), 306–312. 10.18805/lr.v0iOF.3548

[fsn33996-bib-0013] Bilate Daemo, B. , Belew Yohannes, D. , Mulualem Beyene, T. , & Gebreselassie Abtew, W. (2023). AMMI and GGE Biplot analyses for mega environment identification and selection of some high‐yielding cassava genotypes for multiple environments. International Journal of Agronomy, 2023, 1–13. 10.1155/2023/6759698

[fsn33996-bib-0014] Bornhofen, E. , Benin, G. , Storck, L. , Woyann, L. G. , Duarte, T. , Stoco, M. G. , & Marchioro, S. V. (2017). Statistical methods to study adaptability and stability of wheat genotypes. Bragantia, 76, 1–10. 10.1590/1678-4499.557

[fsn33996-bib-0015] Dia, M. , Wehner, T. C. , & Arellano, C. (2016). Analysis of genotype × environment interaction (G9E) using SAS programming. Agronomy Journal, 108, 1838–1852. 10.2134/agronj2016.02.0085

[fsn33996-bib-0016] Eberhart, S. T. , & Russell, W. A. (1966). Stability parameters for comparing varieties 1. Crop Science, 6(1), 36–40. 10.2135/CROPSCI1966.0011183X000600010011X

[fsn33996-bib-0017] Esan, V. I. , Oke, G. O. , Ogunbode, T. O. , & Obisesan, I. A. (2023). AMMI and GGE biplot analyses of Bambara groundnut [*Vigna subterranea* (L.) Verdc.] for agronomic performances under three environmental conditions. Frontiers in Plant Science, 13, 997429. 10.3389/fpls.2022.997429 36743535 PMC9895831

[fsn33996-bib-0018] Farshadfar, E. , Vaisi, Z. , & Yaghotipoor, A. (2011). Non parametric estimation of phenotypic stability in wheat‐barley disomic addition lines. Annals of Biological Research, 2(6), 586–598.

[fsn33996-bib-0019] Gauch, H. G. , & Zobel, R. W. (1996). AMMI analysis of yield trials. In S. Kang & H. Gauch (Eds.), Genotypes by environment interaction (pp. 85–122). CRC Press. 10.1201/9781420049374.ch4

[fsn33996-bib-0020] Gholizadeh, A. , Ghaffari, M. , & Shariati, F. (2021). Use of selection index of ideal genotype (SIIG) in order to select new high yielding sunflower hybrids with desirable agronomic characteristics. Journal of Crop Breeding, 13(38), 116–123. 10.22069/EJCP.2022.19690.2468

[fsn33996-bib-0021] Girgel, U. (2021). Principle component analysis (PCA) of bean genotypes (*Phaseolus vulgaris* L.) concerning agronomic, morphological and biochemical characteristics. Applied Ecology and Environmental Research, 19(3), 1999–2011. 10.15666/aeer/1903_19992011

[fsn33996-bib-0023] Hailemariam Habtegebriel, M. (2022). Adaptability and stability for soybean yield by AMMI and GGE models in Ethiopia. Frontiers in Plant Science, 13, 950992. 10.3389/fpls.2022.950992 36507436 PMC9727298

[fsn33996-bib-0025] Kebede, G. , Worku, W. , Jifar, H. , & Feyissa, F. (2023). Grain yield stability analysis using parametric and nonparametric statistics in oat (*Avena sativa* L.) genotypes in Ethiopia. Grassland Research, 2023, 1–15. 10.1002/glr2.12056

[fsn33996-bib-0026] Khan, M. I. , Kamran Khan, M. , Dagar, V. , Oryani, B. , Akbar, S. S. , Salem, S. , & Dildar, S. M. (2021). Testing environmental Kuznets curve in the USA: What role institutional quality, globalization, energy consumption, financial development, and remittances can play? New evidence from dynamic ARDL simulations approach. Frontiers in Environmental Science, 9, 576. 10.3389/fenvs.2021.789715

[fsn33996-bib-0027] Khan, M. M. H. , Rafii, M. Y. , Ramlee, S. I. , Jusoh, M. , & Al Mamun, M. (2021). AMMI and GGE biplot analysis for yield performance and stability assessment of selected Bambara groundnut (*Vigna subterranea* L. Verdc.) genotypes under the multi‐environmental trials (METs). Scientific Reports, 11(1), 22791. 10.1038/s41598-021-01411-2 34815427 PMC8611061

[fsn33996-bib-0028] Kindie, Y. , Tesso, B. , & Amsalu, B. (2022). AMMI and GGE biplot analysis of genotype by environment interaction and yield stability in early maturing cowpea [*Vigna unguiculata* (L) Walp] landraces in Ethiopia. Plant‐Environment Interactions, 3(1), 1–9. 10.1002/pei3.10068 37283694 PMC10168031

[fsn33996-bib-0029] Kirankumar, R. , Ramesh, S. , Chandana, B. , Basanagouda, G. , Gazala, P. , Siddu, C. , & Kalpana, M. (2023). AMMI model and YREM‐based grain yield stability of horse gram [*Macrotyloma uniflorum* (Lam.) Verdc.] YMV disease resistant genotypes. Mysore Journal of Agricultural Sciences, 57(2), 136–146.

[fsn33996-bib-0030] Kumaresan, D. , & Nadarajan, N. (2010). Genotype × environment interactions for seed yield and its components in sesame (*Sesame indicum* L.). Electronic Journal of Plant Breeding, 1(4), 1126–1132.

[fsn33996-bib-0031] Lee, S. Y. , Lee, H. S. , Lee, C. M. , Ha, S. K. , Park, H. M. , Lee, S. M. , Kwon, Y. , Jeung, J. U. , & Mo, Y. (2023). Multi‐environment trials and stability analysis for yield‐related traits of commercial rice cultivars. Agriculture, 13(2), 256. 10.3390/agriculture13020256

[fsn33996-bib-0032] Lin, C. S. , & Binns, M. R. (1988). A superiority measure of cultivar performance for cultivar × location data. Canadian Journal of Plant Science, 68, 193–198. 10.4141/cjps88-018

[fsn33996-bib-0033] Liu, Z. , Fan, X. , Huang, W. , Yang, J. , Zheng, Y. , Wang, S. , & Qiu, L. (2017). Stability analysis of seven agronomic traits for soybean [*Glycine max* (L.) Merr.] Tokachi nagaha and its derived cultivars using the AMMI model. Plant Production Science, 20(4), 499–506. 10.1080/1343943X.2017.1358095

[fsn33996-bib-0034] Mau, Y. S. , Ndiwa, A. S. , Oematan, S. S. , & Markus, J. E. (2019). Drought tolerance indices for selection of drought tolerant, high yielding upland rice genotypes. Australian Journal of Crop Science, 13(1), 170–178. 10.21475/ajcs.19.13.01.p1778

[fsn33996-bib-0035] Maulana, H. , Dewayani, S. , Solihin, M. A. , Arifin, M. , Amien, S. , & Karuniawan, A. (2020). Yield stability dataset of new orange fleshed sweet potato (*Ipomoea batatas* L. (lam)) genotypes in West Java. Data in Brief, 32, 106297. 10.1016/j.dib.2020.106297 32995393 PMC7502328

[fsn33996-bib-0036] Mirza, M. Y. , Khan, M. A. , Amjad, M. , & Nawaz, M. S. (2013). Stability analysis for economic traits in sesame (*Sesamum indicum* L.). Pakistan Journal of Agricultural, 26(3), 168–177.

[fsn33996-bib-0037] Mirzaei, M. R. , & Hemayati, S. S. (2021). The effect of environment and maternal plant on germination traits of sugar beet seeds and an approach to select the superior genotype. Agricultural Research, 11, 608–614. 10.22092/IJSST.2021.355382.1403

[fsn33996-bib-0038] Mohammadi, R. , & Amri, A. (2008). Comparison of parametric and non‐parametric methods for selecting stable and adapted durum wheat genotypes in variable environments. Euphytica, 159, 419–432. 10.1007/s10681-007-9600-6

[fsn33996-bib-0040] Mwiinga, B. , Sibiya, J. , Kondwakwenda, A. , Musvosvi, C. , & Chigeza, G. (2020). Genotype x environment interaction analysis of soybean (*Glycine max* (L.) Merrill) grain yield across production environments in southern Africa. Field Crops Research, 256, 107922. 10.1016/j.fcr.2020.107922

[fsn33996-bib-0041] Najafi Mirak, T. , Dastfal, M. , Andarzian, B. , Farzadi, H. , Bahari, M. , & Zali, H. (2018). Evaluation of durum wheat cultivars and promising lines for yield and yield stability in warm and dry areas using AMMI model and GGE biplot. Journal of Crop Breeding, 10(28), 1–12. 10.29252/jcb.10.28.1

[fsn33996-bib-0042] Nehbandani, A. , Soltani, A. , Rahemi‐Karizaki, A. , Dadrasi, A. , & Noubakhsh, F. (2021). Determination of soybean yield gap and potential production in Iran using modeling approach and GIS. Journal of Integrative Agriculture, 20(2), 395–407. 10.1016/S2095-3119(20)63180-X

[fsn33996-bib-0043] Oladosu, Y. , Rafii, M. Y. , Abdullah, N. , Magaji, U. , Miah, G. , Hussin, G. , & Ramli, A. (2017). Genotype× environment interaction and stability analyses of yield and yield components of established and mutant rice genotypes tested in multiple locations in Malaysia. Acta Agriculturae Scandinavica Section B Soil and Plant Science, 67(7), 590–606. 10.1080/09064710.2017.1321138

[fsn33996-bib-0044] Olanrewaju, O. S. , Oyatomi, O. , Babalola, O. O. , & Abberton, M. (2021). GGE Biplot analysis of genotype× environment interaction and yield stability in bambara groundnut. Agronomy, 11(9), 1839. 10.3390/agronomy11091839

[fsn33996-bib-0045] Pour‐Aboughadareh, A. , Yousefian, M. , Moradkhani, H. , Poczai, P. , & Siddique, K. H. M. (2019). STABILITYSOFT: A new online program to calculate parametric and non‐parametric stability statistics for crop traits. Applied Plant Science, 7(1), e01211. 10.1002/aps3.1211 PMC6342234 30693157

[fsn33996-bib-0046] Ramzi, E. , Asghari, A. , Khomari, S. , & Mohammaddoust e Chamanabad, H. (2018). Investigation of durum wheat (*Triticum turgidum* L. subsp. durum Desf) lines for tolerance to aluminum stress condition. Journal of Crop Breeding, 10(25), 63–72. 10.29252/jcb.10.25.63

[fsn33996-bib-0047] Rani, M. H. , Begum, S. N. , Khanom, M. S. R. , Rahman, M. H. S. , Hasibuzzaman, A. S. M. , Shugandha, J. N. , Shammy, S. A. , & Akram, M. W. (2021). Genotype‐environment (G× E) interaction, stability and adaptability study on grain yield in advanced rice lines. Bangladesh Journal of Nuclear Agriculture, 35, 9–20.

[fsn33996-bib-0048] Rencher, A. C. , & Christensen, W. F. (2012). Multivariate regression. Methods of multivariate analysis, 3. Scientific Research, 2(6), 586–598. 10.1002/9781118391686

[fsn33996-bib-0049] Ruswandi, D. , Syafii, M. , Maulana, H. , Ariyanti, M. , Indriani, N. P. , & Yuwariah, Y. (2021). GGE biplot analysis for stability and adaptability of maize hybrids in western region of Indonesia. International Journal of Agronomy, 2021, 1–9. 10.1155/2021/2166022

[fsn33996-bib-0050] Ruswandi, D. , Syafii, M. , Wicaksana, N. , Maulana, H. , Ariyanti, M. , Indriani, N. P. , Suryadi, E. , Supriatna, J. , & Yuwariah, Y. (2022). Evaluation of high yielding maize hybrids based on combined stability analysis, sustainability index, and GGE biplot. BioMed Research International, 2022, 1–12. 10.1155/2022/3963850 PMC9343198 35924265

[fsn33996-bib-0051] Samyuktha, S. M. , Malarvizhi, D. , Karthikeyan, A. , Dhasarathan, M. , Hemavathy, A. T. , Vanniarajan, C. , Sheela, V. , Hebziba, S. J. , Pandiyan, M. , & Senthil, N. (2020). Delineation of genotype × environment interaction for identification of stable genotypes to grain yield in mungbean. Frontiers in Agronomy, 2, 577911. 10.3389/fagro.2020.577911

[fsn33996-bib-0052] Santos, A. D. , Amaral Júnior, A. T. , Kurosawa, R. D. N. F. , Gerhardt, I. F. S. , & Fritsche Neto, R. (2017). GGE biplot projection in discriminating the efficiency of popcorn lines to use nitrogen. Agricultural Sciences, 41, 22–31. 10.1590/1413-70542017411030816

[fsn33996-bib-0053] Scavo, A. , Mauromicale, G. , & Ierna, A. (2023). Genotype× environment interactions of potato tuber quality characteristics by AMMI and GGE biplot analysis. Scientia Horticulturae, 310, 111750. 10.1016/j.scienta.2022.111750

[fsn33996-bib-0054] Shojaei, S. H. , Mostafavi, K. , Lak, A. , Omrani, A. , Omrani, S. , Mousavi, S. M. N. , Illes, A. , & Nagy, J. (2021). Evaluation of stability in maize hybrids using univariate parametric methods. Journal of Crop Science and Biotechnology, 1‐8, 269–276. 10.1007/s12892-021-00129-x

[fsn33996-bib-0055] Shukla, G. K. (1972). Some statistical aspects of partitioning genotype‐environmental components of variability. Heredity, 29(2), 237–245. 10.1038/hdy.1972.87 4507945

[fsn33996-bib-0056] Siddquie, M. N. E. A. , & Hoque, M. A. (2023). Genotype× environment interaction on grain yield and yield components in bread wheat. Fundamental and Applied Agriculture, 8(1 & 2), 423–434. 10.5455/faa.146894

[fsn33996-bib-0057] Sinclair, T. R. , Marrou, H. , Soltani, A. , Vadez, V. , & Chandolu, K. C. (2014). Soybean production potential in Africa. Global Food Security, 3(1), 31–40. 10.1016/j.gfs.2013.12.001

[fsn33996-bib-0058] Stansluos, A. A. L. , Öztürk, A. , Niedbała, G. , Türkoğlu, A. , Haliloğlu, K. , Szulc, P. , Omrani, A. , Wojciechowski, T. , & Piekutowska, M. (2023). Genotype–Trait (GT) biplot analysis for yield and quality stability in some sweet corn (*Zea mays* L. saccharata Sturt.). Genotypes Agronomy, 13(6), 1538. 10.3390/agronomy13061538

[fsn33996-bib-0059] Susanto, G. W. A. , Maulana, H. , Putri, P. H. , Purwaningrahayu, R. D. , Wijaya, A. A. , Sekti, B. A. , & Karuniawan, A. (2023). Stability analysis to select the stable and high yielding of black soybean (*Glycine max* (L) Merril) in Indonesia. International Journal of Agronomy, 2023, 1–14. 10.1155/2023/7255444

[fsn33996-bib-0060] Taleghani, D. , Rajabi, A. , Hemayati, S. S. , & Saremirad, A. (2022). Improvement and selection for drought‐tolerant sugar beet (*Beta vulgaris* L.) pollinator lines. *Results* . Engineering, 13, 100367. 10.1016/j.rineng.2022.100367

[fsn33996-bib-0061] Ullah, A. , Shakeel, A. , Ahmed, H. G. M. D. , Naeem, M. , Ali, M. , Shah, A. N. , Wang, L. , Jaremko, M. , Abdelsalam, N. R. , Ghareeb, R. Y. , & Hasan, M. E. (2022). Genetic basis and principal component analysis in cotton (*Gossypium hirsutum* L.) grown under water deficit condition. Frontiers in Plant Science, 13, 981369. 10.3389/fpls.2022.981369 36275586 PMC9583382

[fsn33996-bib-0062] Vaezi, B. , Pour‐Aboughadareh, A. , Mohammadi, R. , Mehraban, A. , Hossein‐Pour, T. , Kooshkan, E. , Ghasemi, S. , Moradkhani, H. , & Siddique, K. (2019). Integrating different stability models to investigate genotype × environment interactions and identify stable and high‐yielding barley genotypes. Euphytica, 215, 1–18. 10.3390/plants12132410

[fsn33996-bib-0063] Vargas, M. , Combs, E. , Alvarado, G. , Atlin, G. , Mathews, K. , & Crossa, J. (2013). META: A suite of SAS programs to analyze multi‐environment breeding trials. Agronomy Journal, 105, 9–11. 10.2134/agronj2012.0016

[fsn33996-bib-0064] Wricke, G. (1962). Übereine Methode zur Erfassung der ökologischen Streubreite in Feldversuchen. Zeitschrift für Pflanzenzüchtung, 47, 92–96.

[fsn33996-bib-0065] Yaghutipoor, A. , Farshadfar, E. , & Saeedi, M. (2017). Investigation of bread wheat genotypes for drought tolerance using suitable combination method. Journal of Environmental Stresses in Crop Sciences, 10, 247–256. 10.22077/escs.2017.581

[fsn33996-bib-0066] Yan, W. , Hunt, L. A. , Sheng, Q. , & Szlavnics, Z. (2000). Cultivar evaluation and mega‐environment investigation based on the GGE biplot. Crop Science, 40(3), 597–605. 10.2135/cropsci2000.403597x

[fsn33996-bib-0067] Yan, W. , Paul, L. , Cornelius, J. C. , & Hunt, L. (2002). Two types of GGE Biplot for analyzing multi‐environmental trial data. Crop Science, 41(3), 656–663. 10.2135/cropsci2001.413656x

[fsn33996-bib-0068] Yan, W. , & Rajcan, I. (2002). Biplot analysis of test sites and trait relations of soybean in Ontario. Crop Science, 42(1), 11–20. 10.2135/cropsci2002.1100 11756248

[fsn33996-bib-0069] Zali, H. , Barati, A. , Pour‐Aboughadareh, A. , Gholipour, A. , Koohkan, S. , Marzoghiyan, A. , Bocianowski, J. , Bujak, H. , & Nowosad, K. (2023). Identification of superior barley genotypes using selection index of ideal genotype (SIIG). Plants, 12(9), 1843. 10.3390/plants12091843 37176901 PMC10181048

[fsn33996-bib-0071] Zhang, P. P. , Hui, S. , Ke, X. W. , Jin, X. J. , Yin, L. H. , Yang, L. , Yang, Q. U. , Wang, S. U. , Feng, N. J. , Diafeng, Z. , & Feng, B. L. (2016). GGE biplot analysis of yield stability and test location representativeness in proso millet (*Panicum miliaceum* L.) genotypes. Journal of Integrative Agriculture, 15(6), 1218–1227. 10.1016/S2095-3119(15)61157-1

